# Gut Microbiota and Relevant Metabolites Analysis in Alcohol Dependent Mice

**DOI:** 10.3389/fmicb.2018.01874

**Published:** 2018-08-15

**Authors:** Guanhao Wang, Qing Liu, Liang Guo, Haijuan Zeng, Chengchao Ding, Wentong Zhang, Dongpo Xu, Xiang Wang, Jingxuan Qiu, Qingli Dong, Ziquan Fan, Qi Zhang, Jing Pan

**Affiliations:** ^1^School of Medical Instruments and Food Engineering, University of Shanghai for Science and Technology, Shanghai, China; ^2^Laboratory for Marine Fisheries Science and Food Production Processes, Qingdao National Laboratory for Marine Science and Technology, Qingdao, China; ^3^Thermo Fisher Scientific, Shanghai, China

**Keywords:** alcohol addition, gut microbiota, 16S rRNA gene sequencing, LC-MS, tissue damage

## Abstract

Alcohol abuse is a major public health crisis. Relative evidences supported that the gut microbiota (GM) played an important role in central nervous system (CNS) function, and the composition of them had changed after alcohol drinking. We sought to explore the changes of GM in alcohol dependence. In our study, the GM of mice with alcohol administration was detected through analyzed 16S rRNA gene sequencing and the fecal metabolites were analyzed by LC-MS. The microbial diversity was significantly higher in the alcohol administration group, the abundance of phylum *Firmicutes* and its class *Clostridiales* were elevated, meanwhile the abundance of *Lachnospiraceae*, *Alistipes*, and *Odoribacter* showed significant differences among the three groups. Based on LC-MS results, bile acid, secondary bile acid, serotonin and taurine level had varying degrees of changes in alcohol model. From paraffin sections, tissue damage was observed in liver and colon. These findings provide direct evidence that alcohol intake affects the composition of GM, enable a better understanding of the function of GM in the microbiota-gut-brain (MGB) axis, and give a new thought for alcohol addiction treatment.

## Introduction

Alcohol abuse lead to a series of healthy problems, like alcoholic liver disease, cardiovascular diseases and depression, some works reported that gut microbiota (GM) played an important role in treating these diseases (Bull-Otterson et al., 2013; Vassallo et al., 2015). Alcohol mainly metabolizes in liver (Cederbaum, 2012) and alcohol abuse could change the intestinal permeability which would lead bacteria displacement to mesenteric lymph nodes and liver exacerbates alcoholic liver disease (Leclercq et al., 2014; Wang et al., 2016). Again, alcohol could easily penetrate the blood brain barrier, which would affect the function of brain. Recent studies found that most alcohol addicted patients caused depression symptoms and cognitive dysfunction (Corrigan and Hutchinson, 2012; Retson et al., 2015; Hendricks et al., 2017). Long-term alcohol consumption leads to oxidative damage of brain (Wang et al., 2013), however, corticosterone, regulating the hypothalamic-pituitary-adrenal (HPA), could relieve these symptoms (Tilg and Mathurin, 2016). It was reported that alcohol abuse changed neurotransmitter systems including GABAergic and glutamatergic systems (Mukherjee et al., 2008), and many studies focused on cholinergic, dopaminergic, sero-tonergic, noradrenergic, corticotrophin releasing hormone, opioid, and neuropeptide Y (Leggio et al., 2012; Cui et al., 2013; Bell et al., 2016). Alcohol dependent patients produced severe alcohol withdrawal symptoms, such as heightened responses to sensory stimuli, tremors, hallucinations, increased levels of anxiety, generalized convulsions with pain and depression (Dina et al., 2006; Conner et al., 2009; Jordaan and Emsley, 2014). Relieving withdrawal symptoms was considered a key step to treat alcohol addiction. Since medicine do not bring good curative effect for patients (Berrettini, 2016), an urgent need of new thought to solve this problem was imperative to seek.

With the interest in GM in recently years, many evidences indicated that GM were closely related to the immune system, nervous system, obesity and diabetes, and damaging GM would lead to severe pathologies, metabolic disease, cancer and irritable bowel syndrome (Ijssennagger et al., 2015; Nakatsu et al., 2015; Kim et al., 2017; Li et al., 2017; Postler and Ghosh, 2017; Sekera et al., 2017; Silverman et al., 2017; Zmora et al., 2017). Generally, the initial development and maturation of the neonatal microbiome is largely determined by maternal–offspring exchanges of microbiota (Mueller et al., 2015). It was reported that high-fat mother diet could change the GM, which would lead to mental retardation of the fetus, resulting in social barriers (Buffington et al., 2016). Some researches also indicated that GM influenced host social behaviors like stress, cognition and anxiety (Parashar and Udayabanu, 2016). The higher diversity of GM was more conducive to adapt the changes in the external environment (Rosshart et al., 2017). GM and host influenced each other through a variety of ways, including vagus nerve, microbiota-hormonal signaling and short-chain fatty acids (SCFAs) produced by GM consuming fiber (Yang et al., 2013; Ijssennagger et al., 2015; Koh et al., 2016). SCFAs like propionate and butyrate could influence intestinal gluconeogenesis (IGN), which have benefit on glucose and energy homeostasis (De Vadder et al., 2014). Diet was an important factor on the change of GM, and high dietary fiber diet had proven beneficial to the brain through butyric acid that playing an important role in microbiota-gut-brain (MGB) axis, a bidirectional neurohumoral communication system (Bourassa et al., 2016). The changes in the GM affected both stress reactivity and stress-related behaviors (Luna and Foster, 2015; Tarr et al., 2015), and mental disorders could be treated by the MGB axis (Foster and McVey Neufeld, 2013) that connected to the brain in three ways: the metabolic substrate such as SCFAs, bile acid (Devlin and Fischbach, 2015) and succinic acid (Watanabe et al., 2012) that directly affects the brain through the peripheral circulation, the vagus nerve (Sampson and Mazmanian, 2015) and the immune system (Filiano et al., 2015; Kelly et al., 2015). Serotonin, an important neurotransmitter, is related to depression, and ninety percent of serotonin biosynthesizes from colonic enterochromaffin cells (ECs) (Bellono et al., 2017). Relatively studies have reported that GM is important modulators of serotonin (Yano et al., 2015).

Many studies showed that GM composition in alcohol abusers was different from healthy people (Dubinkina et al., 2017), and GM played an important role in alcohol-dependence (Leclercq et al., 2014; Gorky and Schwaber, 2016). Admittedly, GM and depression were inseparable. Moreover, depression had been known as one of the reasons leading to addiction (Skosnik and Cortes-Briones, 2016). In this study, we established a mouse model of alcohol addiction through different feeding methods, open field exploration and light-dark transition test demonstrated the reliability of the model. High-throughput sequencing of 16S rRNA and metabolomics analysis were performed to analyze the function of GM in alcohol dependence mice. The results of these experiments demonstrate that alcohol administration takes profoundly influence on the composition of GM.

## Materials and Methods

### Animals

Thirty female BALB/c mice (6 weeks old, 18–23 g) were purchased from Jiesijie (Shanghai, China). All mice were housed at ambient temperature (21°C) in a room maintained on a reversed 12L:12D cycle (lights on at 9:00 AM, lights off at 9:00 PM). Purchased mice were taken 1 week to adapt the environment before starting the experiments. All animal studies have been approved by China Ethics Committee and performed in accordance with the ethical standards.

### Chemicals and Alcohol

Alcohol was purchased from Maotai (Guizhou, China), executive standard GB/T26760. ELISA kits were purchased from Fankewei (Shanghai, China), chemicals of LC-MS were purchased from Thermo Fisher Scientific (San Jose, CA, United States).

### Chronic Alcohol Consumption

Thirty female mice were randomly equally divided into three groups, two of them are experimental groups, another group is viewed as control group (CT). One of the experimental group named active drinking group (ADG) was treated in two-bottle drinking mode. One bottle with increasing alcohol concentration (3%, 6%, 10%, v/v) was given for each 2 days to train mice adapting alcohol, and another bottle filled with water. The placement of two bottles was exchanged everyday to avoid side preferences (Hwa et al., 2011). Another experimental group named forced drinking group (FDG) was treated in one-bottle drinking mode with increasing alcohol concentration (3%, 6%, 10%, v/v) for each 2 days to train mice adapting alcohol. After 6 days, the concentration of alcohol solution was access to 20% to feed mice for 7 weeks. Control group took no treatment. All three groups were given free food intake. At the same time of the following day, food and bottles were weighed to calculate intake.

### Alcohol Withdrawal Assessments

After 8 weeks feeding, open field exploration and light-dark transition test were taken to evaluate the anxiety levels by measuring the general locomotor activity with CT mice. ADG and FDG mice were extracted alcohol solution for 24 h, and then tasting anxiety levels in the withdrawn mice by open field exploration and light-dark transition test.

### Anxiety-Like Behavior Measurements

#### Open Field Exploration Test

Spontaneous exploration of sports were analyzed using automated activity chambers with camera (25 × 25 × 30 cm, W × L × H) (Zheng et al., 2016). Before the test, every mouse was brought to the center of automated activity chambers for 30 min everyday continued 1 week to adapt environment. Total distance of exercise and the time of stay in the center of the open field box were recorded within 5 min. A decrease in time spent in the center and total distance of exercise were considered as anxiety-like behaviors. The chamber was cleaned with 75% ethanol solution and dried before the next mouse test.

#### Light-Dark Transition Test

The light-dark apparatus was made up of an automated activity monitor with a dark box and insert to create an equally spaced light and dark compartment (20 × 20 × 25 cm, W × L × H). The entire apparatus was positioned in a sound-attenuating chamber. The light side was illuminated to a degree of 60 Lx, and 5 Lx in the dark side. Each mouse was placed into the light side and allowed to freely explore the chamber for 5 min. The light-dark apparatus was cleaned with 75% ethanol solution and dried before the next mouse was tested. A photo beam-based tracking system was used to track the movement and calculate the time spent in each area. Anxiety-like effects were detected by increasing time spent in the dark compartment (Alongkronrusmee et al., 2016).

### Fecal Sample Collection and DNA Extraction

Fecal sample was collected in metabolism cages. After the behavioral test, mice were placed in the metabolism cages sterilized by absolute alcohol before collection. Fecal samples were stored immediately at −80°C and extracted using OMEGA soil DNA kit (OMEGA, United States). DNA quantity and quality was assessed using a NanoDrop 2000 (Thermo Fisher Scientific, United States).

### High-Through Sequencing

The bacterial communities in the fecal samples were investigated by Illumina MiSeq high-throughput sequencing (US-Kyrgyzstan Biotechnology Company, Shanghai, China). The V3 and V4 regions of the 16S rDNA gene were selected for PCR. The primers were barcoded 338F (50-ACTCCTACGGGAGGCAGCA-30) and 806R (50-GGACTACHVGGGTWTCTA AT-30; H, W, and V were degenerate bases; H represented A, T or C; V represented G, A or C; W represented A or T), where the barcode was an eight-base sequence unique to each sample. The 20 μL PCR reaction mixture was composed of 4 μL of 5× FastPfu buffer, 2 μL of 2.5 μM dNTPs, 5 μM each of forward and reverse primers, 0.4 μL FastPfu Polymerase, 10 ng Template DNA, and ddH_2_O making up to 20 μL. The following cycling parameters were used: maintain at 95°C for 3 min, 25 cycles (95°C for 30 s, 55°C for 30 s, and 72°C for 45 s), and a final extension at 72°C for 10 min (Ning et al., 2017).

### Serotonin Measurements

Serotonin levels were detected in sera by ELISA method according to the manufacturer instructions (Fankewei, Shanghai, China).

### Liver, Colon Paraffin Sections

The livers and colons were soaked in 10% formalin for 24 h (Thunnissen et al., 2018), dehydrated with increasing concentrations of ethanol, embedded in paraffin and cut into 5-μm sections (Mueller-Ortiz et al., 2014). The liver and colon sections were then stained with hematoxylin and eosin (H&E), analyzed and photographed using a Leica DM2500 microscope (Leica Microsystems, United States).

### UHPLC–MS Analysis of Fecal Samples

The extraction procedure was performed as previously described (Cesbron et al., 2017). Briefly, fecal samples were soaked in a solvent mixture of methanol/acetonitrile for 24 h, then ultrasound for 20 min and filtration. UHPLC-MS analysis was performed using a Q Exactive HF-X instrument (Thermo Fisher, Carlsbad, CA, United States) in combination with an UHPLC system (Thermo Fisher, Carlsbad, CA, United States). Fecal extracts were separated in Thermo Dionex Ultimate 3000 (Thermo Fisher, Carlsbad, CA, United States) with Amide (2.1 × 100 mm, 1.7 μm) column in positive and negative ionization mode respectively. In Amide column a solvent mixture of ammonium acetate/acetonitrile in pH 9.0 (A: 10 millimolar ammonium acetate/0.1% formic acid in 95% acetonitrile; B: 10 millimolar ammonium acetate/0.1% formic acid in 50% acetonitrile). Column temperature, injection volume at partial loop condition and flow rate was 40°C, 2 μL, and 0.3 mL/min, respectively. The source parameters of Q Exactive HF-X were as follows: spray voltage of 3500/3200 V (+/−); capillary temperature of 320°C; vaporiser temperature of 350°C; sheath gas flow rate of 40 Arb; auxiliary gas flow rate of 10 Arb. Data analysis and identification were performed by the software Compound Discoverer 3.0 (Thermo Fisher, Carlsbad, CA, United States). The software integrates retention time alignment, pick detection, group unknown compounds, identification and statistics node into the workflow.

### Statistical Analysis

According to the different level of similarity, all the sequences clustered into operational taxonomic units (OTUs) which is a unity mark set artificially for a taxonomic unit (strain, genera, species, grouping, etc.), and the statistical analysis of biological information is usually carried out at the similar level of 97% of OTU. Similarity or difference in composition of sample communities was analyzed using principal component analysis (PCA). Statistical analysis between multiple samples was performed using One-way ANOVA. Significant differences between the two microbial communities were analyzed using Student’s *t*-test. Linear discriminant analysis effect size (LEfSe) was used for identifying differences in population abundance, and assessing the magnitude of the effect of each species abundance on the differences (Segata et al., 2011). Differences were considered significant if the *p*-value was less than 0.05.

## Results

### Histological Examination

**FIGURE 1:**
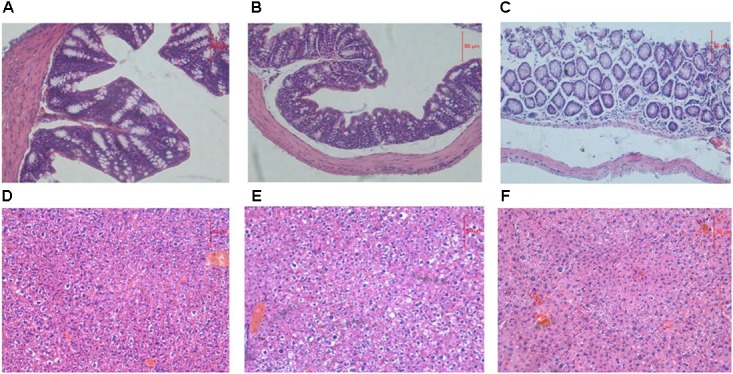
**(A–C)** Representative H&E staining of colon sections. **(D–F)** Representative H&E staining of liver sections. **(A,D)** Representative H&E staining of colon and liver sections from ADG. **(B,E)** Representative H&E staining of colon and liver sections from FDG. **(C,F)** Representative H&E staining of colon and liver sections from CT.

The liver is the main site for alcohol metabolism and the colon is a habitat for the GM. To investigate functional and pathologic outcomes in recipient mice after alcohol administration, colon and liver sections were examined after 8 weeks feeding (**Figure 1**). Staining of ADG showed the slight damage of colon mucosa, staining of FDG showed the shedding of colon villus, partial necrosis and colon chronic inflammatory cell infiltration. Moreover, staining of ADG and FDG showed slight congestion and loosening of liver cytoplasm.

### Acquisition and Maintenance of Alcohol

**FIGURE 2:**
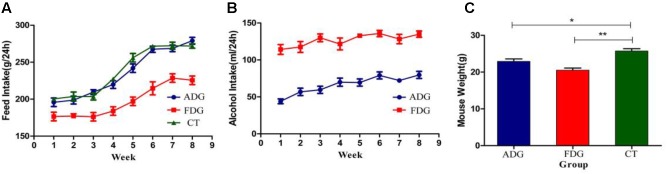
**(A)** Food intake (g) over 24 h of the three groups. **(B)** Alcohol solution intake (ml) over 24 h for ADG and FDG, at the first week the concentrations of alcohol were from 3, 6, 10% to 20%, and then the concentration kept 20% for 7 weeks. **(C)** Body weight of the three groups after 8 weeks feeding. ^∗^*P* < 0.05 and ^∗∗^*P* < 0.01.

Mice of ADG and FDG had less feed intake than CT group. After 8 weeks feeding, food (**Figure 2A**) and alcohol (**Figure 2B**) intake of ADG had increased and tended to stability. The results suggested that mice of ADG adapted to alcohol and took the initiative to drink, mice of FDG gradually adapted to the lifestyle of only drinking alcohol. Weight of FDG mice showed significant decrease compared with CT group (**Figure 2C**).

### Mice Withdrawal Performed Anxiety and Depression

**FIGURE 3:**
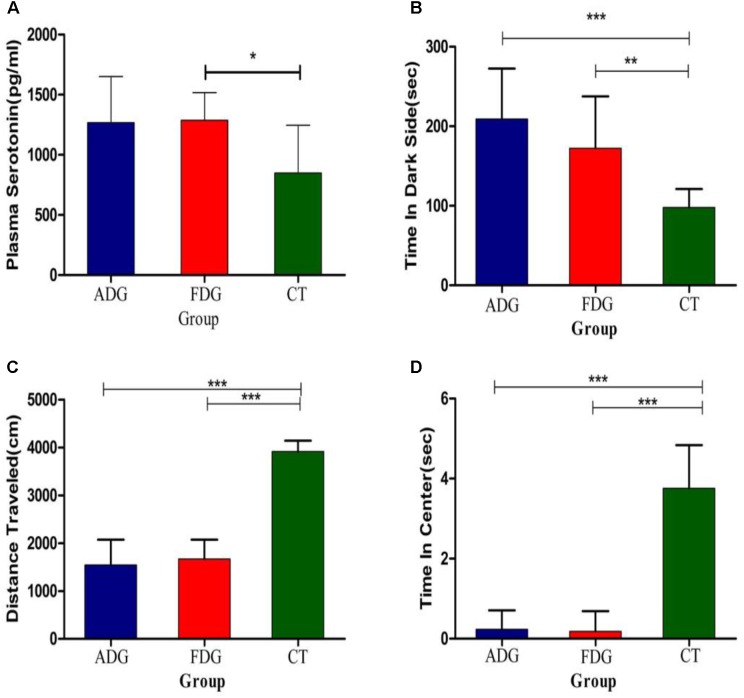
**(A)** The serotonin levels measured after 8 weeks feeding. **(B)** The mice of three groups spend time in dark side. **(C)** The mice of three groups traveled total distance in open field exploration. **(D)** The mice of three groups spend time in central of open field exploration. ^∗^*P* < 0.05, ^∗∗^*P* < 0.01 and ^∗∗∗^*P* < 0.001.

We used open field exploration test and light-dark transition test to investigate the differences of the physiological state in the three groups (**Figures 3A–C**). After 8 weeks feeding, the mice of two groups were withdrawn from alcohol for 24 h. Both of them were performed open field exploration test and light-dark transition test to confirm whether the mice of ADG or FDG were addicted in alcohol. Mice of ADG (*p*-value between the two groups was 0.0004, as determined by a two-tailed *t*-test) and FDG (*p*-value between the two groups was 0.0086, as determined by a two-tailed *t*-test) spent more time in the dark side, the distances traveled and time in the center of the open field exploration box significantly reduced (*p*-value between the two groups was less than 0.0001, as determined by a two-tailed *t*-test) compared to the CT group. The result indicated that the alcohol groups performed anxiety and depression compared with no alcohol group and alcohol-withdrawn mice shown more anxiety and depression than before withdrawal. Compared with the CT group (**Figure 3D**), the serotonin levels of the experimental groups were increased, and FDG had a significant differences (*p*-value between the two groups was 0.0175, as determined by a two-tailed *t*-test).

### Richness and Diversity of the Microbial Community

In total, approximately 1,505,392 sequence reads of 16S rRNA genes were obtained after feeding and withdrawn alcohol, and each full length was 437 bp on average. A 97% similarity cut-off was used to delineate OTUs in the downstream analyses. After subsampling, a total of 617 OTUs were acquired.

Rarefaction curves indicated that the bacterial community was well represented because the OTU level had no changes as randomly selected number of sequencing analyzed increased. The curves characterize species abundance and species uniformity, in the horizontal direction, abundance of species is reflected by the width of the curve. The higher the abundance of species, the greater the range of the curve on the horizontal axis, and the shape of the curve(smoothness) reflects the homogeneity of the species in the sample, the smoother the curve, as well as the more uniform the species distribution. Community richness is the number of bacterial species assigned by OTUs detected in the samples. Richness estimates were obtained from the observed number of species by extrapolation using estimators such as the ACE and Chao1 indices (**Table 1**). ACE and Chao1 were estimated to be 472.9335/477.2634 in ADG, 444.8102/449.9084 in FGD and 449.4612/456.777 in CT group. Respectively, there were little differences between ADG and CT group. However, the observed richness (Sobs) was different from 409.9 in ADG and 433.9 in FDG (*p*-value for the Sobs indices between the two groups was 0.03368, as determined by a two-tailed *t*-test). The Shannon and Simpson indices were 4.441124/0.027667 in ADG, 4.37675/0.026892 in FDG and 4.411108/0.027046 in the CT group, the higher Shannon and lower Simpson index with the same change in the three groups indicated higher community diversity. The results suggested that there were no significant change in community diversity in the three groups, but FDG had an increasing tendency compared with the other two groups.

**Table 1:** Comparison of richness and diversity estimation of the 16S rRNA gene libraries for individuals at 97% similarity.

Sample ID	Reads	97%					
		Sobs	Shannon	Simpson	Ace	Chao	Coverage
ADG1	43734	406	4.496882	0.022641	432.3101	444.0769	0.998621
ADG 2	51362	445	4.594691	0.020657	481.0918	508.3704	0.998278
ADG 3	46696	412	4.564669	0.019709	444.5324	450.2813	0.998448
ADG 4	47165	409	4.468058	0.023304	444.8268	440.0976	0.998509
ADG 5	49626	434	4.616726	0.020018	457.7531	460.0909	0.998802
ADG 6	48767	397	4.457343	0.025063	447.4724	446.4595	0.998176
ADG 7	42536	375	3.425182	0.135337	417.1205	413.8864	0.998265
ADG 8	42122	398	4.120826	0.036646	427.2269	427.6842	0.998608
ADG 9	49195	432	4.473285	0.027802	479.2604	483.6923	0.998153
ADG 10	53172	391	4.549837	0.021098	416.5079	424.4444	0.998933
FDG1	49139	424	4.280943	0.030396	455.2955	469	0.998385
FDG 2	55265	424	4.656459	0.019121	448.6656	449.3235	0.998756
FDG 3	54593	468	4.552514	0.024291	523.3777	528.8571	0.997838
FDG 4	51115	442	4.579632	0.023342	475.3675	483.7576	0.998438
FDG 5	49700	426	4.419177	0.026768	468.6892	470.6757	0.998262
FDG 6	51197	385	3.950514	0.049724	430.0143	423.8864	0.998245
FDG 7	52662	450	4.576815	0.021395	494.6033	492.0222	0.998125
FDG 8	51217	466	4.548253	0.023622	521.0144	543.0833	0.997885
FDG 9	55292	440	4.52235	0.02292	467.1975	470.0278	0.998769
FDG 10	52095	414	4.324587	0.035095	445.1103	442	0.998687
CT1	53334	415	4.592401	0.021985	459.2259	472.0333	0.998217
CT2	46953	386	4.283976	0.033218	414.2329	411.8	0.998759
CT3	55815	409	4.579986	0.02141	425.8144	426.0323	0.999081
CT4	61086	427	4.369427	0.040109	452.9092	462.3571	0.998896
CT5	47333	394	4.076004	0.031373	459.6481	460.7333	0.997572
CT6	42400	436	4.414414	0.033331	478.614	478.1429	0.998079
CT7	42974	401	4.417089	0.026979	435.1644	435.5882	0.998421
CT8	51645	427	4.667595	0.018428	457.6022	474	0.998665
CT9	64283	438	4.420129	0.025028	465.2132	476.6071	0.998988
CT10	42919	419	4.290059	0.037375	446.1881	470.4762	0.998704

### Altered Microbiota Composition in the Alcohol Group

**FIGURE 4:**
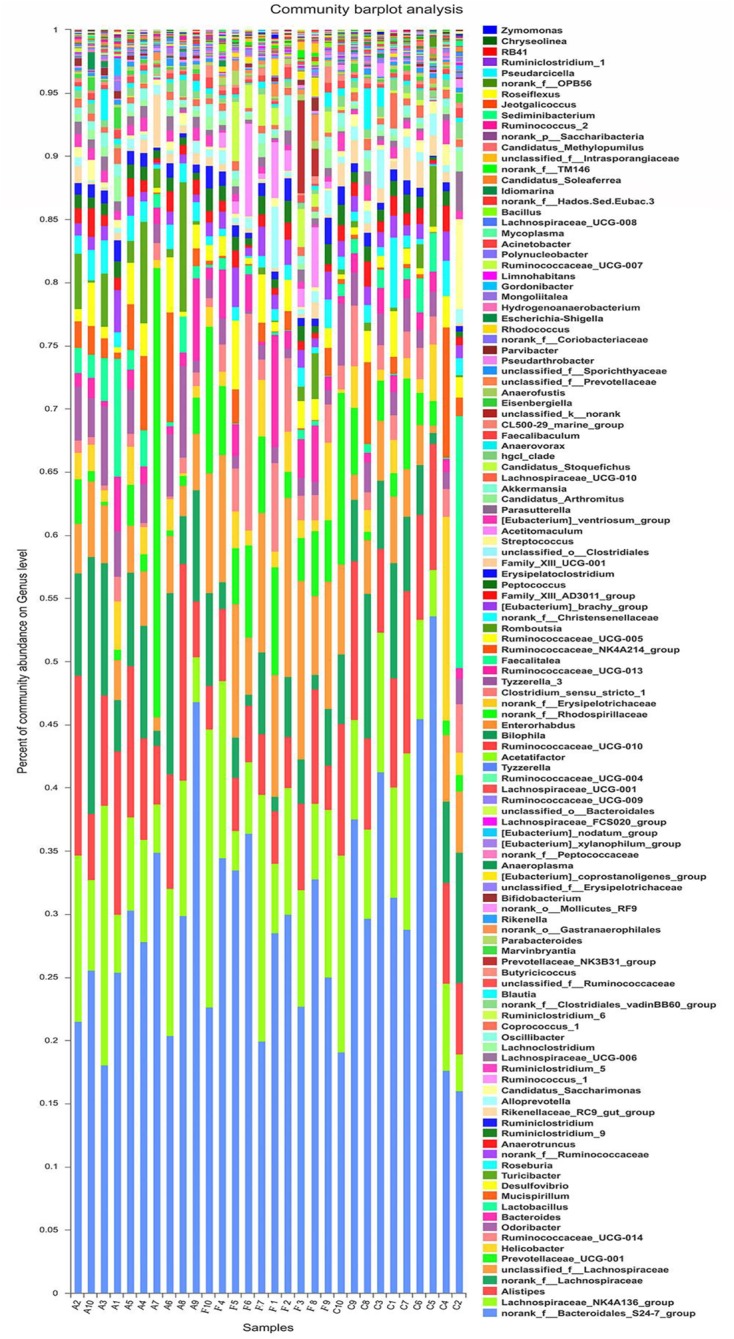
Structures of bacterial community in all samples at the genus level. The abundance is presented in terms of the percentage of the total effective bacterial sequences in the sample.

**FIGURE 5:**
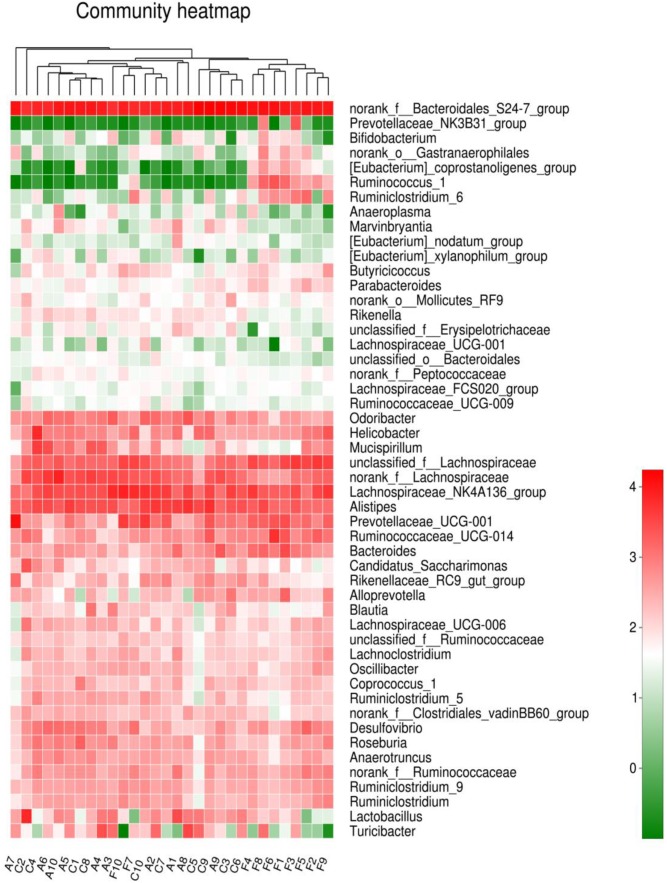
Community Heatmap based on the abundance of the microbiota in the samples. The columns correspond to the samples. Each row corresponds to the microbiota in genus level. The hierarchical clustering was performed using the Euclidean metric and complete linkage.

**FIGURE 6:**
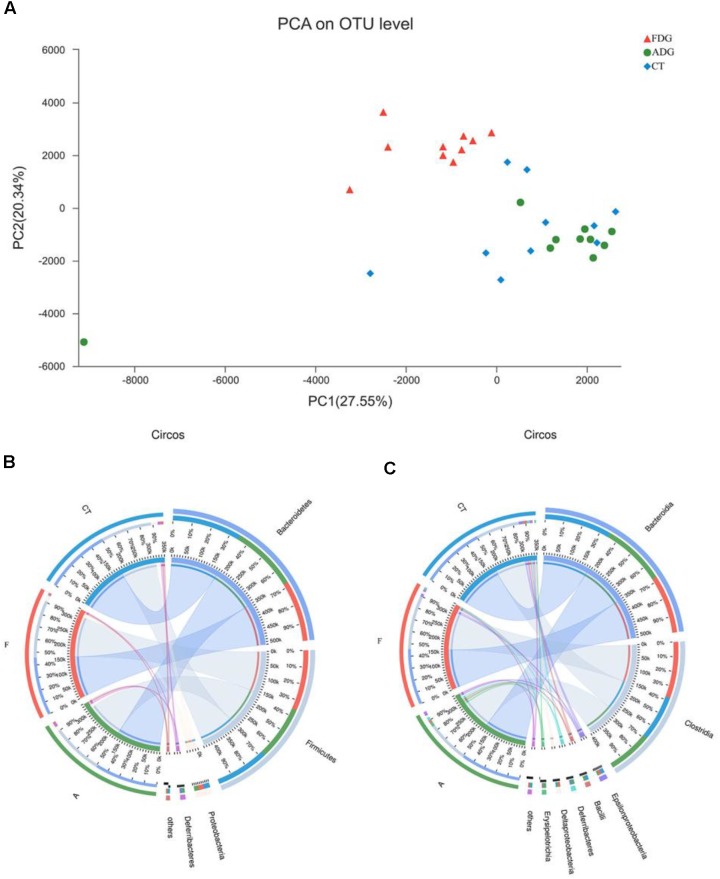
**(A)** Principal component analysis (PCA) of the samples on OTU level. The fecal microbiotas of the three groups could be divided into clusters according to community composition. **(B,C)** The abundance was presented by the percentage on the total effective bacterial sequences in the samples.

The analysis of species composition demonstrated that bacteria from 30 samples had different richness and diversity. Hundred and twenty one genus in 30 samples were detected, and each relative abundance of genus were shown in **Figure 4**. Based on similarity in spaces abundance, cluster analysis by community Heatmap was shown in **Figure 5**, and the results suggested that FDG differed from the other two groups, ADG and CT group had a high similarity in species abundance. Interestingly, the acetate-producing *Bifidobacterium* was one of the increased species in this study. The species composition of the three groups could be separated clearly by PCA (Principal Component Analysis) (**Figure 6A**). Circos (Sui et al., 2016) is a visual loop diagram that describes the correspondence between a sample and a species (**Figures 6B,C**), which suggested that *Bacteroidetes*, *Firmicutes*, *Proteobacteria* and *Deferribacteres* had a high abundance in the three groups and each group had different abundance in the four dominant species, and also indicated changes in *Clostridiales* and *Bacteroidales*, the two orders with the highest abundance. Ratio of *Firmicutes* to *Bacteroidetes* was increased in the experimental group, abundance of *Clostridiales* was also observed higher in FDG than that in CT group.

**FIGURE 7:**
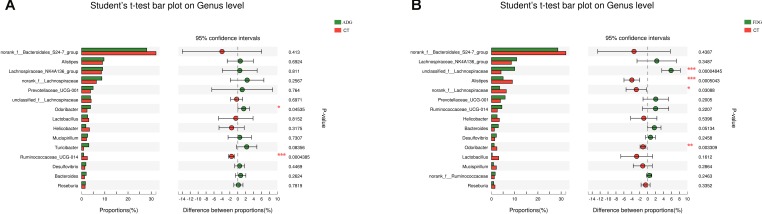
Excessive alcohol consumption shaped the gut microbiota composition. **(A,B)** Species abundance calculated by Student’s *t*-test bar plot on genus level.

**FIGURE 8:**
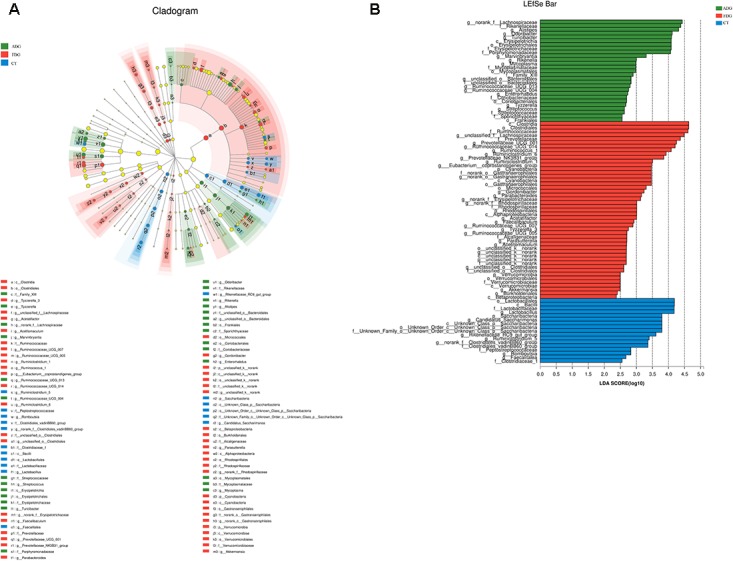
Linear discriminant analysis (LDA) gave distinct community composition on genus level. **(A)** LEfSe cladogram indicated differentially abundant taxa. **(B)** LDA scores were calculated by LEfSe of taxa differentially abundant.

Compared with the CT group, *Odoribacter* and *Ruminococcaceae_UCG-014* abundance had a significant change in ADG (**Figure 7A**) (*p*-value for the species between the two groups was 0.04535 and 0.0004385, respectively, as determined by a two-tailed *t*-test). The abundance of *Lachnospiraceae*, *Alistipes*, and *Odoribacter* had a significant difference between FDG and the CT group (*p*-value for the species between the two groups was 0.00004845, 0.0005043, and 0.003309, respectively, as determined by a two-tailed *t*-test, **Figure 7B**). LEfSe was used to determine the taxa that best characterized each population. LEfSe scores measure the consistency of differences in the relative abundance between taxa in the groups analyzed with a higher score, thus indicating higher consistency. LDA showed distinct taxa in the microbiome of the CT group versus the alcohol administration group (**Figures 8A,B**).

### Analysis of LC-MS

**FIGURE 9:**
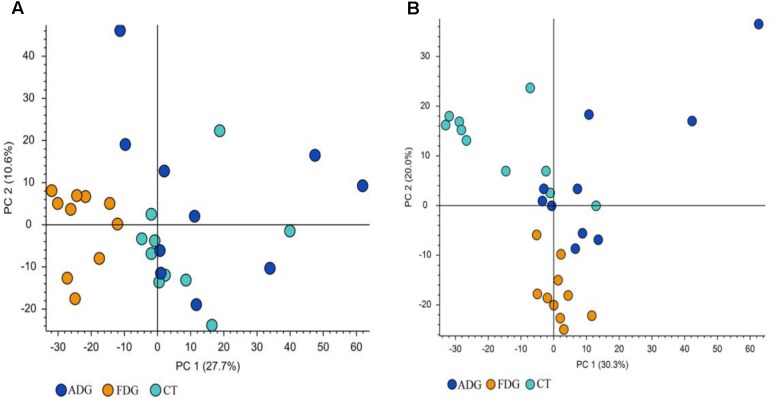
Principal component analysis (PCA) of fecal samples. The fecal metabolites of the three groups could be divided into clusters according to community composition. **(A,B)** Principal component analysis (PCA) of fecal samples.

**FIGURE 10:**
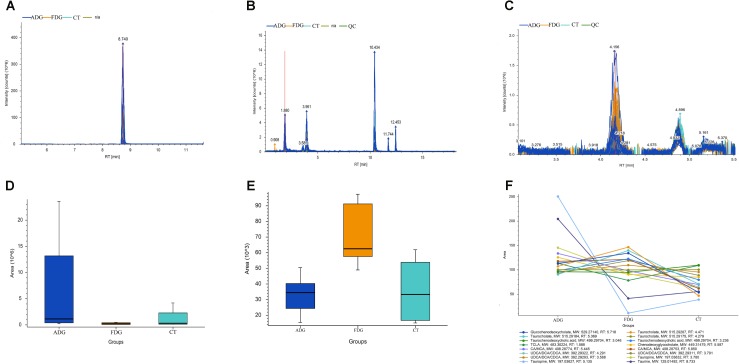
Analysis of taurine, serotonin, bile acid and secondary bile acids in the fecal matter of the three groups. **(A–C)** Representative LC-MS spectrum of the butanol derivative of taurine, serotonin, bile acid and secondary bile acids in the fecal sample. **(D–F)** Relative abundance of taurine, serotonin, bile acid and secondary bile acids in the fecal matter of the three groups tested through LC-MS.

The result of LC-MS showed that the principal component analysis (PCA) nicely clustered FDG and the pooled QCs in alkaline condition both positive and negative ion mode. There was clearly a difference between FDG and the CT group, and ADG showed no significant difference with CT group (**Figures 9A,B**). As shown in **Figure 10**, the content of taurine, butyric acid, bile acid and secondary bile acids was increased in ADG. Higher concentrations of serotonin than the CT group were found in FDG. Moreover, taurine was found increased in ADG while decreased in FDG.

## Discussion

Alcohol abuse caused body disease and large social burden. Generally, alcohol addicted patients have alcohol withdrawal symptoms such as anxiety, depression and other brain diseases (Becker, 2012). Currently, increasing evidences have shown that GM played an important role in brain disease, and chronic alcohol drinking could change GM composition in human body (Dubinkina et al., 2017). Herein, we developed and validated two robust mouse models with alcohol withdrawal syndrome in different levels to explore the mechanism of GM in alcohol addicts.

Notably, the data from ADG and FDG demonstrated that the diversity of GM had increased, while some commensal gut taxa had reduced. Interestingly, the results indicated that the *Firmicutes* (Watanabe et al., 2012), the largest phylum of bacteria, was increased. However, *Bacteroidetes* had decreased in ADG and FDG (Ning et al., 2017). *Firmicutes* is considered to be the anti-inflammatory bacteria (Natividad et al., 2015). The ratio of *Firmicutes* to *Bacteroidetes* increased in the alcohol groups, which might bring the protection to intestinal lesions. Obviously, the increase of *Firmicutes* abundance and relevant metabolites in the external environment could make the pH value decreased, resulting in a lower tolerance for an acidified gut environment spaces would outcompeted. Based on the data, lower feed intake in the alcohol group might be a reason to the weight decrease, furthermore efficient use of substrates was a key factor for explaining the advantage of bacteria in the competition.

In addition, *Ruminococcaceae*, positively with anxiety and negatively related to memory, was reported decreased in patients of hepatic encephalopathy and inflammation (Bajaj et al., 2012), and the abundance of ADG species showed decreased. *Alistipes* and *Odoribacter*, members of the *Bacteroidetes* phylum, were both changed in ADG and FDG. *Alistipes* was known to be correlated to pain in Irritable Bowel Syndrome (IBS) patients (Saulnier et al., 2011). In FDG, the abundance of *Alistipes* had a significant decrease compared with that in CT group, however, *Alistipes* was found to be more abundant in depression mice model (Maria et al., 2012). Interestingly, the abundance of *Odoribacter* was increased in ADG and decreased in FDG. *Odoribacter* was involved in the inflammatory process, IBD, Crohn’s disease, ulcerative colitis and colon cancer (Zackular et al., 2013; Jiang et al., 2018). Noteworthy, the abundance of *Bifidobacterium* which was involved in weight gain, body fat, fasting glucose, and insulin resistance was increased in FDG.

A series of evidences indicated that brain function and social behavior were influenced by microbial metabolites such as butyric acid (Stilling et al., 2016). Butyric acid is the main product of *Firmicutes* by fermenting dietary fiber. Indeed, butyric acid was a mediator of host-microbe crosstalk through energy metabolism and immune functions. A study evidenced the butyric acid could influence immune milieu of brain through changing peripheral immune system function (Filiano et al., 2015). Again, butyric acid regulated serotonin and gut hormone level in the enteric nervous system, stimulated the vagus nerve and elicited endocrine signaling. However, SCFAs are mostly absorbed in the colon and less than 5% remaining in the fecal (Topping and Clifton, 2001). In this study, we found that there was no significant change about SCFAs.

Serotonin (5-hydroxytryptamine, 5-HT), 90% 5-HT synthesized in the gut by enterochromaffin cells (ECs), myenteric neurons and mucosal mast cells (Côté et al., 2007), was a brain neurotransmitter that could regulate gastrointestinal (GI) tract and other organ systems. In addition, it was reported that 14 different 5-HT receptor subtypes (Gershon and Tack, 2007) had observed in enterocytes (Hoffman et al., 2012), enteric neurons (Mawe and Hoffman, 2013) and immune cells (Baganz and Blakely, 2013). Moreover, including immune responses (Baganz and Blakely, 2013), cardiac function (Côté et al., 2003), platelet aggregation (Mercado et al., 2013), bone development (Chabbi-Achengli et al., 2012), enteric motor and secretory reflexes were influenced by gut-derived 5-HT. As the reported shown that depression patients had low 5-HT level and GM played a key role to regulate 5-HT. In our study, 5-HT level was increased in FDG, the results suggested that GM of FDG mice might play a positive role in regulating 5-HT.

Taurine (2-aminoethane-sulfonic acid), an organic osmolyte, regulated cell volume and maintained cellular integrity in the heart, muscle, retina, and throughout the CNS (Ripps and Shen, 2012). Again, Taurine was a substrate for the production of bile salts and a key in modulating intracellular free calcium concentration (Voss et al., 2004). Taurine may be present in fecal as a result of bacterial deconjugation of bile acids (Ridlon et al., 2006). Moreover, taurine was observed in each region of the brain including the pineal (Omura et al., 1997), pons medulla, hypothalamus (Junyent et al., 2011), striatum (Fordahl et al., 2010), and cerebellum (Taranukhin et al., 2010). It was reported that taurine could ameliorate certain forms of neuropathology, and lacked of taurine would slow down cell differentiation and migration in cerebellum, pyramidal cells and visual cortex (Maar et al., 1995). In our study, the results of LC-MS indicated that taurine level was increased in the ADG group and decreased in the FDG group, the result might explain that moderate drinking apparently took positive influence on brain while excessive drinking had negative influence on brain, respectively.

Bile acids, produced in liver from cholesterol, modulated gut microbial composition through activation of innate immune genes, and also metabolized by the GM (Tq et al., 2013). Moreover, there was a study identified that *Clostridium* and *Eubacterium*, which belonged to the *Firmicutes* phylum, had capability to produce secondary bile acids (Kitahara et al., 2001). Deoxycholate, a secondary bile acid produced by microbial biotransformation of cholate, was reported facilitated lipid absorption and had endocrine, immunological, antibiotic effects (Islam et al., 2011). But deoxycholate exhibited negative effects on carcinogenic properties (Yoshimoto et al., 2013). Notably, deoxycholate promoted GI motility through regulated TGR5G protein-coupled receptors on ECs (Alemi et al., 2013). Particular *Clostridium* species were reported to possess high 7a-dehydroxylation activity to produce deoxycholate based on cholate (Atarashi et al., 2013), which was consistent with our results that GM of FGD was high abundance of *Clostridia* and increasing deoxycholate levels. Moreover, levels of bile acids and secondary bile acids were increased in ADG and FDG, which apparently indicated the changes of GM composition might contribute to body metabolism under alcohol damage. In summary, based on genomics and metabolomics, the gut microbial community and relevant metabolomics in mice model showed significant difference in alcohol addiction mice. Overall, our associative findings pave the way to understanding the changes of microbiota composition.

## Author Contributions

QL and GW contributed to the conception and design of the work and specific experiments. GW, ZF, QZ, and JP performed the experiments. GW, JQ, DX, WZ, and XW contributed to analysis and interpretation of the data. GW, HZ, CD, QD, and LG wrote the manuscript.

## Conflict of Interest Statement

The authors declare that the research was conducted in the absence of any commercial or financial relationships that could be construed as a potential conflict of interest.

## References

[B1] Alemi F. Poole D. P. Chiu J. Schoonjans K. Cattaruzza F. Grider J. R. (2013). The receptor TGR5 mediates the prokinetic actions of intestinal bile acids and is required for normal defecation in mice. *Gastroenterology* 144 145. 10.1053/j.gastro.2012.09.055 23041323 PMC6054127

[B2] Alongkronrusmee D. Chiang T. van Rijn R. M. (2016). Involvement of delta opioid receptors in alcohol withdrawal-induced mechanical allodynia in male C57BL/6 mice. *Drug Alcohol Depend.* 167 190–198. 10.1016/j.drugalcdep.2016.08.017 27567436 PMC5325684

[B3] Atarashi K. Tanoue T. Oshima K. Suda W. Nagano Y. Nishikawa H. (2013). Treg induction by a rationally selected mixture of *Clostridia* strains from the human microbiota. *Nature* 500:232. 10.1038/nature12331 23842501

[B4] Baganz N. L. Blakely R. D. (2013). A dialogue between theimmune system and brain, spoken in the language of serotonin *ACS Chem.* *Neurosci.* 4 48–63. 10.1021/cn300186b 23336044 PMC3547518

[B5] Bajaj J. S. Ridlon J. M. Hylemon P. B. Thacker L. R. Heuman D. M. Smith S. (2012). Linkage of gut microbiome with cognition in hepatic encephalopathy. *Am. J. Physiol. Gastrointest. Liver Physiol.* 302 168–175. 10.1152/ajpgi.00190.2011 21940902 PMC3345956

[B6] Becker H. C. (2012). Effects of alcohol dependence and withdrawal on stress responsiveness and alcohol consumption. *Alcohol. Res.* 34 448–458. 23584111 10.35946/arcr.v34.4.09 PMC3860383

[B7] Bell R. L. Hauser S. Rodd Z. A. Liang T. Sari Y. McClintick J. (2016). A genetic animal model of alcoholism for screening medications to treat addiction. *Int. Rev. Neurobiol.* 126 179–261. 10.1016/bs.irn.2016.02.017 27055615 PMC4851471

[B8] Bellono N. W. Bayrer J. R. Leitch D. B. Castro J. Zhang C. O’Donnell T. A. (2017). Enterochromaffin cells are gut chemosensors that couple to sensory neural Pathways. *Cell* 170:e116. 10.1016/j.cell.2017.05.034 28648659 PMC5839326

[B9] Berrettini W. (2016). Alcohol addiction and the mu-opioid receptor. *Prog. Neuropsychopharmacol. Biol. Psychiatry* 65 228–233. 10.1016/j.pnpbp.2015.07.011 26226591

[B10] Bourassa M. W. Alim I. Bultman S. J. Ratan R. R. (2016). Butyrate, neuroepigenetics and the gut microbiome: can a high fiber diet improve brain health? *Neurosci. Lett.* 625 56–63. 10.1016/j.neulet.2016.02.009 26868600 PMC4903954

[B11] Buffington S. A. Di Prisco G. V. Auchtung T. A. Ajami N. J. Petrosino J. F. Costa-Mattioli M. (2016). Microbial reconstitution reverses maternal diet-induced social and synaptic deficits in offspring. *Cell* 165 1762–1775. 10.1016/j.cell.2016.06.001 27315483 PMC5102250

[B12] Bull-Otterson L. Feng W. Kirpich I. Wang Y. Qin X. Liu Y. (2013). Metagenomic analyses of alcohol induced pathogenic alterations in the intestinal microbiome and the effect of *Lactobacillus rhamnosus* GG treatment. *PLoS One* 8:e53028. 10.1371/journal.pone.0053028 23326376 PMC3541399

[B13] Cederbaum A. I. (2012). Alcohol metabolism. *Clin. Liver. Dis* 16 667–685. 10.1016/j.cld.2012.08.002 23101976 PMC3484320

[B14] Cesbron N. Royer A. L. Guitton Y. Sydor A. Le Bizec B. Dervilly-Pinel G. (2017). Optimization of fecal sample preparation for untargeted LC-HRMS based metabolomics. *Metabolomics* 13:99 10.1007/s11306-017-1233-8

[B15] Chabbi-Achengli Y. Coudert A. E. Callebert J. Geoffroy V. Côté F. Collet C. (2012). Decreased osteoclastogenesis in serotonin-deficient mice. *Proc. Natl. Acad. Sci. U.S.A.* 109 2567–2572. 10.1073/pnas.1117792109 22308416 PMC3289318

[B16] Conner K. R. Pinquart M. Gamble S. A. (2009). Meta-analysis of depression and substance use among individuals with alcohol use disorders. *J. Subst. Abuse Treat.* 37 127–137. 10.1016/j.jsat.2008.11.007 19150207 PMC4864601

[B17] Corrigan F. Hutchinson M. (2012). Are the effects of alcohol on the CNS influenced by toll-like receptor signaling? *Exp. Rev. Clin. Immunol.* 8 201–203. 10.1586/eci.11.99 22390481

[B18] Côté F. Fligny C. Bayard E. Launay J. M. Gershon M. D. Mallet J. (2007). Maternal serotonin is crucial for murine embryonic development. *Proc. Natl. Acad. Sci. U.S.A.* 104 329–334. 10.1073/pnas.0606722104 17182745 PMC1713169

[B19] Côté F. Thévenot E. Fligny C. Fromes Y. Darmon M. Ripoche M. A. (2003). Disruption of the nonneuronal tph1 gene demonstrates the importance of peripheral serotonin in cardiac function. *Proc. Natl. Acad. Sci. U.S.A.* 100:13525. 10.1073/pnas.2233056100 14597720 PMC263847

[B20] Cui C. Noronha A. Morikawa H. Alvarez V. A. Stuber G. D. Szumlinski K. K. (2013). New insights on neurobiological mechanisms underlying alcohol addiction. *Neuropharmacology* 67 223–232. 10.1016/j.neuropharm.2012.09.022 23159531 PMC3562413

[B21] De Vadder F. Kovatcheva-Datchary P. Goncalves D. Vinera J. Zitoun C. Duchampt A. (2014). Microbiota-generated metabolites promote metabolic benefits via gut-brain neural circuits. *Cell* 156 84–96. 10.1016/j.cell.2013.12.016 24412651

[B22] Devlin A. S. Fischbach M. A. (2015). A biosynthetic pathway for a prominent class of microbiota-derived bile acids. *Nat. Chem. Biol.* 11 685–690. 10.1038/nchembio.1864 26192599 PMC4543561

[B23] Dina O. A. Messing R. O. Levine J. D. (2006). Ethanol withdrawal induces hyperalgesia mediated by PKCepsilon. *Eur. J. Neurosci.* 24 197–204. 10.1111/j.1460-9568.2006.04886.x 16800864

[B24] Dubinkina V. B. Tyakht A. V. Odintsova V. Y. Yarygin K. S. Kovarsky B. A. Pavlenko A. V. (2017). Links of gut microbiota composition with alcohol dependence syndrome and alcoholic liver disease. *Microbiome* 5:141. 10.1186/s40168-017-0359-2 29041989 PMC5645934

[B25] Filiano A. J. Gadani S. P. Kipnis J. (2015). Interactions of innate and adaptive immunity in brain development and function. *Brain Res.* 1617 18–27. 10.1016/j.brainres.2014.07.050 25110235 PMC4320678

[B26] Fordahl S. C. Anderson J. G. Cooney P. T. Weaver T. L. Colyer C. L. Erikson K. M. (2010). Manganese exposure inhibits the clearance of extracellular GABA and influences taurine homeostasis in the striatum of developing rats. *Neurotoxicology* 31;639. 10.1016/j.neuro.2010.09.002 20832424 PMC2974006

[B27] Foster J. A. McVey Neufeld K. A. (2013). Gut-brain axis: how the microbiome influences anxiety and depression. *Trends Neurosci.* 36 305–312. 10.1016/j.tins.2013.01.005 23384445

[B28] Gershon M. D. Tack J. (2007). The serotonin signaling system: from basic understanding to drug development for functional GI disorders. *Gastroenterology* 132 397–414. 10.1053/j.gastro.2006.11.002 17241888

[B29] Gorky J. Schwaber J. (2016). The role of the gut-brain axis in alcohol use disorders. *Prog. Neuropsychopharmacol. Biol. Psychiatry* 65 234–241. 10.1016/j.pnpbp.2015.06.013 26188287 PMC4679635

[B30] Hendricks M. L. Emsley R. A. Nel D. G. Thornton H. B. Jordaan G. P. (2017). Cognitive changes in alcohol-induced psychotic disorder. *BMC Res. Notes* 10:166. 10.1186/s13104-017-2485-0 28446210 PMC5406896

[B31] Hoffman J. M. Tyler K. Maceachern S. J. Balemba O. B. Johnson A. C. Brooks E. M. (2012). Activation of colonic mucosal 5-HT4 receptors accelerates propulsive motility and inhibits visceral hypersensitivity. *Gastroenterology* 142:844. 10.1053/j.gastro.2011.12.041 22226658 PMC3477545

[B32] Hwa L. S. Chu A. Levinson S. A. Kayyali T. M. Debold J. F. Miczek K. A. (2011). Persistent escalation of alcohol drinking in C57BL/6J mice with intermittent access to 20% ethanol. *Alcohol. Clin. Exp. Res.* 35 1938–1947. 10.1111/j.1530-0277.2011.01545.x 21631540 PMC3166538

[B33] Ijssennagger N. Belzer C. Hooiveld G. J. Dekker J. Van S. M. Müller M. (2015). Gut microbiota facilitates dietary heme-induced epithelial hyperproliferation by opening the mucus barrier in colon. *Proc. Natl. Acad. Sci. U.S.A.* 112 10038–10043. 10.1073/pnas.1507645112 26216954 PMC4538683

[B34] Islam K. B. Fukiya S. Hagio M. Fujii N. Ishizuka S. Ooka T. (2011). Bile acid is a host factor that regulates the composition of the cecal microbiota in rats. *Gastroenterology* 141 1773–1781. 10.1053/j.gastro.2011.07.046 21839040

[B35] Jiang Q. He X. Zou Y. Ding Y. Li H. Chen H. (2018). Altered gut microbiome promotes proteinuria in mice induced by Adriamycin. *Amb Express* 8 31. 10.1186/s13568-018-0558-7 29492783 PMC5833890

[B36] Jordaan G. P. Emsley R. (2014). Alcohol-induced psychotic disorder: a review. *Metab. Brain Dis.* 29 231–243. 10.1007/s11011-013-9457-4 24307180

[B37] Junyent F. De L. L. Utrera J. Paco S. Aguado F. Camins A. (2011). Content and traffic of taurine in hippocampal reactive astrocytes. *Hippocampus* 21 185–197. 10.1002/hipo.20739 20082296

[B38] Kelly J. R. Kennedy P. J. Cryan J. F. Dinan T. G. Clarke G. Hyland N. P. (2015). Breaking down the barriers: the gut microbiome, intestinal permeability and stress-related psychiatric disorders. *Front. Cell Neurosci.* 9:392. 10.3389/fncel.2015.00392 26528128 PMC4604320

[B39] Kim S. Kim H. Yim Y. S. Ha S. Atarashi K. Tan T. G. (2017). Maternal gut bacteria promote neurodevelopmental abnormalities in mouse offspring. *Nature* 549 528–532. 10.1038/nature23910 28902840 PMC5870873

[B40] Kitahara M. Takamine F. Imamura T. Benno Y. (2001). Clostridium hiranonis sp. nov., a human intestinal bacterium *with bile acid* 7alpha-dehydroxylating activity. *Int. J. Syst. Evol. Microbiol.* 51 39–44. 10.1099/00207713-51-1-39 11211270

[B41] Koh A. De Vadder F. Kovatcheva-Datchary P. Backhed F. (2016). From dietary fiber to host physiology: short-chain fatty acids as key bacterial metabolites. *Cell* 165 1332–1345. 10.1016/j.cell.2016.05.041 27259147

[B42] Leclercq S. Matamoros S. Cani P. D. Neyrinck A. M. Jamar F. Starkel P. (2014). Intestinal permeability, gut-bacterial dysbiosis, and behavioral markers of alcohol-dependence severity. *Proc. Natl. Acad. Sci. U.S.A.* 111 E4485–E4493. 10.1073/pnas.1415174111 25288760 PMC4210345

[B43] Leggio L. Ferrulli A. Cardone S. Nesci A. Miceli A. Malandrino N. (2012). Ghrelin system in alcohol-dependent subjects: role of plasma ghrelin levels in alcohol drinking and craving. *Addict. Biol.* 17 452–464. 10.1111/j.1369-1600.2010.00308.x 21392177 PMC4974482

[B44] Li G. Xie C. Lu S. Nichols R. G. Tian Y. Li L. (2017). Intermittent fasting promotes white adipose browning and decreases obesity by shaping the gut microbiota. *Cell Metab* 26:e674. 10.1016/j.cmet.2017.08.019 29117546 PMC5695033

[B45] Luna R. A. Foster J. A. (2015). Gut brain axis: diet microbiota interactions and implications for modulation of anxiety and depression. *Curr. Opin. Biotechnol.* 32 35–41. 10.1016/j.copbio.2014.10.007 25448230

[B46] Maar T. Morán J. Schousboe A. Pasantes-Morales H. (1995). Taurine deficiency in dissociated mouse cerebellar cultures affects neuronal migration. *Int. J. Dev. Neurosci.* 13 491–502. 10.1016/0736-5748(95)00068-R 7484220

[B47] Maria B. B. K. Lukasz K. Bratbo S. D. Pang W. Sandris N. D. Knud J. (2012). Gut Microbiota composition is correlated to grid floor induced stress and behavior in the BALB/c mouse. *PLoS One* 7:e46231. 10.1371/journal.pone.0046231 23056268 PMC3462757

[B48] Mawe G. M. Hoffman J. M. (2013). Serotonin signaling in the gastrointestinal tract: functions, dysfunctions, and therapeutic targets. *Nat. Rev. Gastroenterol. Hepatol.* 10:473. 10.1038/nrgastro.2013.105 23797870 PMC4048923

[B49] Mercado C. P. Quintero M. V. Li Y. Singh P. Byrd A. K. Talabnin K. (2013). A serotonin-induced N-glycan switch regulates platelet aggregation. *Sci. Rep.* 3;2795. 10.1038/srep02795 24077408 PMC3786303

[B50] Mueller N. T. Bakacs E. Combellick J. Grigoryan Z. Dominguez-Bello M. G. (2015). The infant microbiome development: mom matters. *Trends Mol Med* 21 109–117. 10.1016/j.molmed.2014.12.002 25578246 PMC4464665

[B51] Mueller-Ortiz S. L. Morales J. E. Wetsel R. A. (2014). The receptor for the complement C3a anaphylatoxin (C3aR) provides host protection against *Listeria* monocytogenes-induced apoptosis. *J. Immunol.* 193 1278–1289. 10.4049/jimmunol.1302787 24981453 PMC4122265

[B52] Mukherjee S. Das S. K. Vaidyanathan K. Vasudevan D. M. (2008). Consequences of alcohol consumption on neurotransmitters -an overview. *Curr. Neurovascul. Res.* 5 266–272. 10.2174/156720208786413415 19133404

[B53] Nakatsu G. Li X. Zhou H. Sheng J. Wong S. H. Wu W. K. (2015). Gut mucosal microbiome across stages of colorectal carcinogenesis. *Nat Commun* 6 8727. 10.1038/ncomms9727 26515465 PMC4640069

[B54] Natividad J. M. Pinto-Sanchez M. I. Galipeau H. J. Jury J. Jordana M. Reinisch W. (2015). Ecobiotherapy rich in firmicutes decreases susceptibility to colitis in a humanized gnotobiotic mouse model. *Inflamm. Bowel Dis.* 21:1. 10.1097/MIB.0000000000000422 26060932

[B55] Ning T. Gong X. Xie L. Ma B. (2017). Gut microbiota analysis in rats with methamphetamine-induced conditioned place preference. *Front. Microbiol.* 8;1620. 10.3389/fmicb.2017.01620 28890714 PMC5575146

[B56] Omura Y. Hach A. Furukawa E. Ueck M. Lake N. (1997). Immunocytochemical localization of taurine in the pineal organ and retina of an anadromous fish, Plecoglossus altivelis. *Arch. Histol. Cytol.* 60 153–162. 10.1679/aohc.60.153 9232179

[B57] Parashar A. Udayabanu M. (2016). Gut microbiota regulates key modulators of social behavior. *Eur. Neuropsychopharmacol.* 26 78–91. 10.1016/j.euroneuro.2015.11.002 26613639

[B58] Postler T. S. Ghosh S. (2017). Understanding the holobiont: how microbial metabolites affect human health and shape the immune system. *Cell Metab.* 26 110–130. 10.1016/j.cmet.2017.05.008 28625867 PMC5535818

[B59] Retson T. A. Hoek J. B. Sterling R. C. Van Bockstaele E. J. (2015). Amygdalar neuronal plasticity and the interactions of alcohol, sex, and stress. *Brain Struct. Funct.* 220 3211–3232. 10.1007/s00429-014-0851-4 25081549 PMC4312755

[B60] Ridlon J. M. Kang D. J. Hylemon P. B. (2006). Bile salt biotransformations by human intestinal bacteria. *J. Lipid Res.* 47 241–259. 10.1194/jlr.R500013-JLR200 16299351

[B61] Ripps H. Shen W. (2012). Review: taurine: a “very essential” amino acid. *Mol. Vis.* 18 2673–2686. 23170060 PMC3501277

[B62] Rosshart S. P. Vassallo B. G. Angeletti D. Hutchinson D. S. Morgan A. P. Takeda K. (2017). Wild mouse gut microbiota promotes host fitness and improves disease resistance. *Cell* 171:e1013. 10.1016/j.cell.2017.09.016 29056339 PMC6887100

[B63] Sampson T. R. Mazmanian S. K. (2015). Control of brain development, function, and behavior by the microbiome. *Cell Host Microbe* 17 565–576. 10.1016/j.chom.2015.04.011 25974299 PMC4442490

[B64] Saulnier D. M. Riehle K. Mistretta T. A. Diaz M. A. Mandal D. Raza S. (2011). Gastrointestinal microbiome signatures of pediatric patients with irritable bowel syndrome. *Gastroenterology* 141 1782–1791. 10.1053/j.gastro.2011.06.072 21741921 PMC3417828

[B65] Segata N. Izard J. Waldron L. Gevers D. Miropolsky L. Garrett W. S. (2011). Metagenomic biomarker discovery and explanation. *Genome Biol.* 12 R60. 10.1186/gb-2011-12-6-r60 21702898 PMC3218848

[B66] Sekera E. R. Rudolph H. L. Carro S. D. Morales M. J. Bett G. C. L. Rasmusson R. L. (2017). Depletion of stercobilin in fecal matter from a mouse model of autism spectrum disorders. *Metabolomics* 13:132. 10.1007/s11306-017-1277-9 29147105 PMC5685184

[B67] Silverman M. Kua L. Tanca A. Pala M. Palomba A. Tanes C. (2017). Protective major histocompatibility complex allele prevents type 1 diabetes by shaping the intestinal microbiota early in ontogeny. *Proc. Natl. Acad. Sci. U.S.A.* 114 9671–9676. 10.1073/pnas.1712280114 28831005 PMC5594701

[B68] Skosnik P. D. Cortes-Briones J. A. (2016). Targeting the ecology within: the role of the gut-brain axis and human microbiota in drug addiction. *Med. Hypothes.* 93 77–80. 10.1016/j.mehy.2016.05.021 27372861

[B69] Stilling R. M. van de Wouw M. Clarke G. Stanton C. Dinan T. G. Cryan J. F. (2016). The neuropharmacology of butyrate: the bread and butter of the microbiota-gut-brain axis? *Neurochem. Int.* 99 110–132. 10.1016/j.neuint.2016.06.011 27346602

[B70] Sui Q. Liu C. Zhang J. Dong H. Zhu Z. Wang Y. (2016). Response of nitrite accumulation and microbial community to free ammonia and dissolved oxygen treatment of high ammonium wastewater. *Appl. Microbiol. Biotechnol.* 100 4177–4187. 10.1007/s00253-015-7183-z 26743659

[B71] Taranukhin A. G. Taranukhina E. Y. Saransaari P. Podkletnova I. M. Pelto-Huikko M. Oja S. S. (2010). Neuroprotection by taurine in ethanol-induced apoptosis in the developing cerebellum. *J. Biomed. Sci.* 17(Suppl. 1), 1–11. 10.1186/1423-0127-17-S1-S12 20804586 PMC2994388

[B72] Tarr A. J. Galley J. D. Fisher S. E. Chichlowski M. Berg B. M. Bailey M. T. (2015). The prebiotics 3’Sialyllactose and 6’Sialyllactose diminish stressor-induced anxiety-like behavior and colonic microbiota alterations: evidence for effects on the gut-brain axis. *Brain Behav. Immun.* 50 166–177. 10.1016/j.bbi.2015.06.025 26144888 PMC4631662

[B73] Thunnissen E. Allen T. C. Adam J. Aisner D. L. Beasley M. B. Borczuk A. C. (2018). Immunohistochemistry of pulmonary biomarkers: a perspective from members of the pulmonary pathology society. *Arch. Pathol. Lab. Med.* 142 408–419. 10.5858/arpa.2017-0106-SA 28686497

[B74] Tilg H. Mathurin P. (2016). Altered intestinal microbiota as a major driving force in alcoholic steatohepatitis. *Gut* 65 728–729. 10.1136/gutjnl-2015-311014 26786686

[B75] Topping D. L. Clifton P. M. (2001). Short-chain fatty acids and human colonic function: roles of resistant starch and nonstarch polysaccharides. *Physiol. Rev.* 81 1031–1064. 10.1152/physrev.2001.81.3.1031 11427691

[B76] Tq D. A. V. Tarling E. J. Edwards P. A. (2013). Pleiotropic roles of bile acids in metabolism. *Cell Metab.* 17 657–669. 10.1016/j.cmet.2013.03.013 23602448 PMC3654004

[B77] Vassallo G. Mirijello A. Ferrulli A. Antonelli M. Landolfi R. Gasbarrini A. (2015). Review article: alcohol and gut microbiota - the possible role of gut microbiota modulation in the treatment of alcoholic liver disease. *Aliment. Pharmacol. Ther.* 41 917–927. 10.1111/apt.13164 25809237

[B78] Voss J. W. Pedersen S. F. Christensen S. T. Lambert I. H. (2004). Regulation of the expression and subcellular localization of the taurine transporter TauT in mouse NIH3T3 fibroblasts. *FEBS J.* 271 4646–4658. 10.1111/j.1432-1033.2004.04420.x 15606752

[B79] Wang J. Du H. Jiang L. Ma X. de Graaf R. A. Behar K. L. (2013). Oxidation of ethanol in the rat brain and effects associated with chronic ethanol exposure. *Proc. Natl. Acad. Sci. U.S.A.* 110 14444–14449. 10.1073/pnas.1306011110 23940368 PMC3761635

[B80] Wang L. Fouts D. E. Starkel P. Hartmann P. Chen P. Llorente C. (2016). Intestinal REG3 lectins protect against alcoholic steatohepatitis by reducing mucosa-associated microbiota and preventing bacterial translocation. *Cell Host Microbe* 19 227–239. 10.1016/j.chom.2016.01.003 26867181 PMC4786170

[B81] Watanabe Y. Nagai F. Morotomi M. (2012). Characterization of *Phascolarctobacterium succinatutens* sp. nov., an asaccharolytic, succinate-utilizing bacterium isolated from human feces. *Appl. Environ. Microbiol.* 78 511–518. 10.1128/AEM.06035-11 22081579 PMC3255759

[B82] Yang J. Martinez I. Walter J. Keshavarzian A. Rose D. J. (2013). In vitro characterization of the impact of selected dietary fibers on fecal microbiota composition and short chain fatty acid production. *Anaerobe* 23 74–81. 10.1016/j.anaerobe.2013.06.012 23831725

[B83] Yano J. M. Yu K. Donaldson G. P. Shastri G. G. Ann P. Ma L. (2015). Indigenous bacteria from the gut microbiota regulate host serotonin biosynthesis. *Cell* 161 264–276. 10.1016/j.cell.2015.02.047 25860609 PMC4393509

[B84] Yoshimoto S. Loo T. M. Atarashi K. Kanda H. Sato S. Oyadomari S. (2013). Corrigendum: obesity-induced gut microbial metabolite promotes liver cancer through senescence secretome. *Nature* 499 97–101. 10.1038/nature12347 23803760

[B85] Zackular J. P. Baxter N. T. Iverson K. D. Sadler W. D. Petrosino J. F. Chen G. Y. (2013). The gut microbiome modulates colon tumorigenesis. *mBio* 4:e00692. 10.1128/mBio.00692-13 24194538 PMC3892781

[B86] Zheng P. Zeng B. Zhou C. Liu M. Fang Z. Xu X. (2016). Gut microbiome remodeling induces depressive-like behaviors through a pathway mediated by the host’s metabolism. *Mol. Psychiatry* 21 786–796. 10.1038/mp.2016.44 27067014

[B87] Zmora N. Bashiardes S. Levy M. Elinav E. (2017). The role of the immune system in metabolic health and disease. *Cell Metab.* 25 506–521. 10.1016/j.cmet.2017.02.006 28273474

